# Supplementary material for: Exploring the Expression of Cardiac Regulators in a Vertebrate Extremophile: The Cichlid Fish Oreochromis (Alcolapia) alcalica
Source: J Dev Biol. 2020 Oct 4;8(4):22. doi: 10.3390/jdb8040022 (PMC7712675; doi:10.3390/jdb8040022)
Supplement: Supplementary file 1 [file jdb-08-00022-s001.pdf]

SUPPLEMENTARY MATERIALS

Supplementary Movies ***(scan QR codes to link to youtube videos)***

Contracting heart during *O. alcalica* development.

A Early pharyngula stage *O. alcalica* embryo at 66 hours post fertilisation (hpf) with contracting linear heart tube. Lateral view; anterior to the top left. **B** 3 days post fertilisation (dpf) pharyngula stage *O. alcalica* embryo with contracting linear heart tube and early vascularisation. Dorsal view; anterior to the left. **C** 4dpf pharyngula stage *O. alcalica* embryo with looping and contracting heart tube. Lateral view; anterior to the lower left. **D** 5dpf hatched *O. alcalica* with two-chambered heart. Lateral view; anterior to the left.

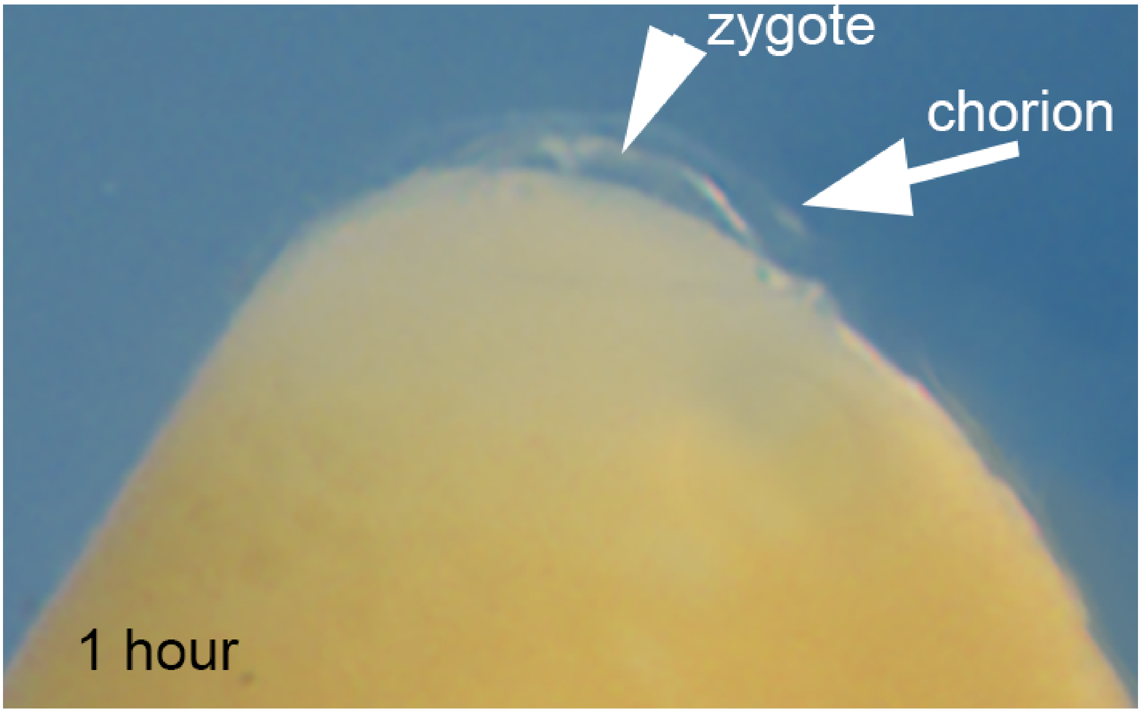

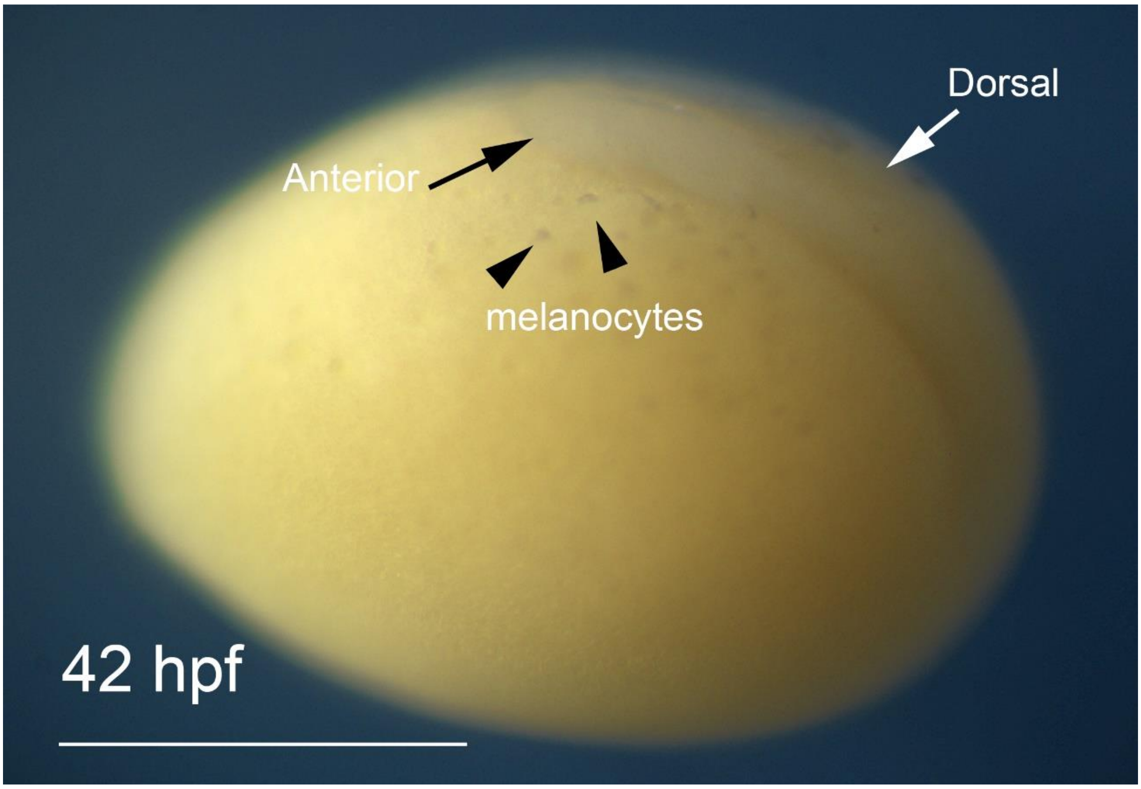

Supplemental Figure 1 Enlarged view of Figure 1A, and a labelled version of Figure 1E.
Top: The chorion (a membrane surrounding the one-cell embryo/zygote and its yolk) is indicated. **Bottom**: The axis running along the dorsal side of the embryo is indicated; anterior is revealed by the emerging pigment cells/melanocytes.

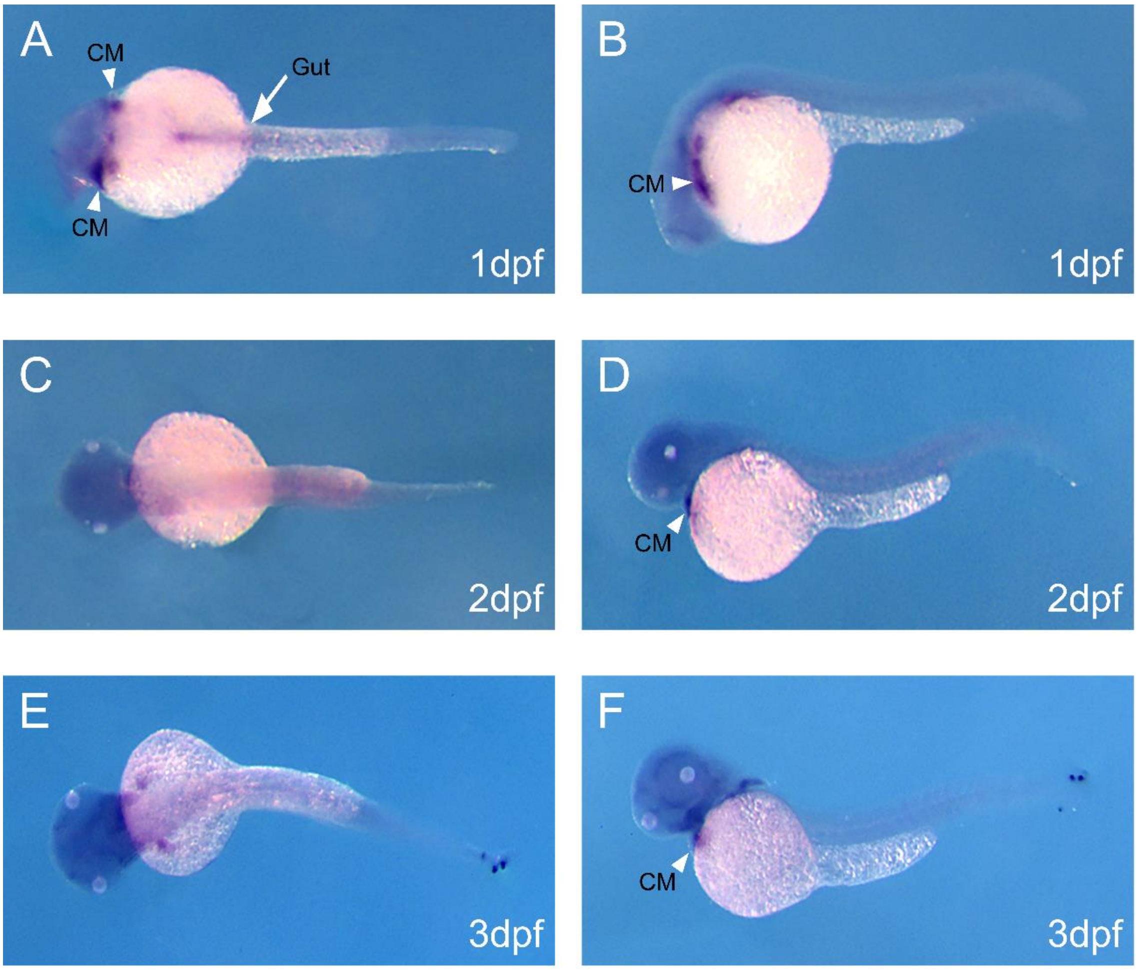

Supplementary Figure 2. Expression of cardiac regulator *gata4* in zebrafish.

A-F *in situ* hybridisation of *gata4* in zebrafish from 1-3 days post fertilisation (dpf). At 1dpf *gata4* is expressed in cardiomyocytes (CM) located in bilateral fields of the anterior lateral plate mesoderm and in the endodermal-derived gut (**A, B**). At 2dpf and 3dpf *gata4*-expressing cardiomyocytes (CM) are located at the midline (**C-F**). White arrowheads denote cardiomyocytes. White arrows mark the gut. Dorsal views; anterior to the left (**A, C, E**). Lateral views; anterior to the left (**B, D, F**).

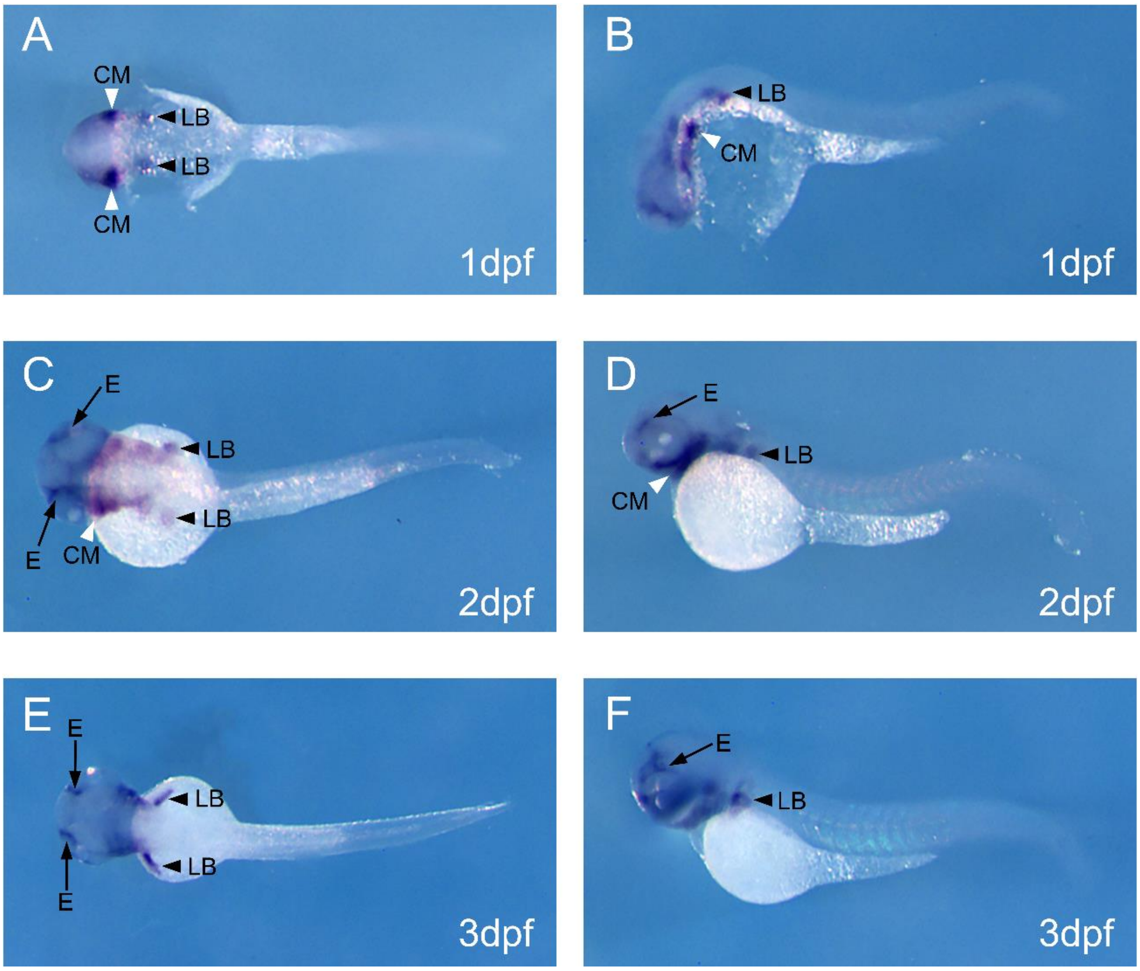

Supplementary Figure 3. Expression of cardiac regulator *tbx5b* in zebrafish.

A-F *in situ* hybridisation of *tbx5b* in zebrafish from 1-3dpf. At 1dpf *tbx5b* is expressed in cardiomyocytes located in bilateral fields of the anterior lateral plate mesoderm (**A, B**). At 2dpf, *tbx5b* is expressed in cardiomyocytes at the midline (**C, D**). *tbx5b* expression is also detected in the limb bud from 1-3dpf (**A-F**) and in the eye at 2dpf and 3dpf (**C-F**). White arrowheads denote cardiomyocytes. Black arrowheads mark limb buds. Black arrows denote the eyes. Dorsal views; anterior to the left (**A, C, E**). Lateral views; anterior to the left (**B, D, F**). Abbreviations: CM, cardiomyocytes; LB, limb bud; E, eye.

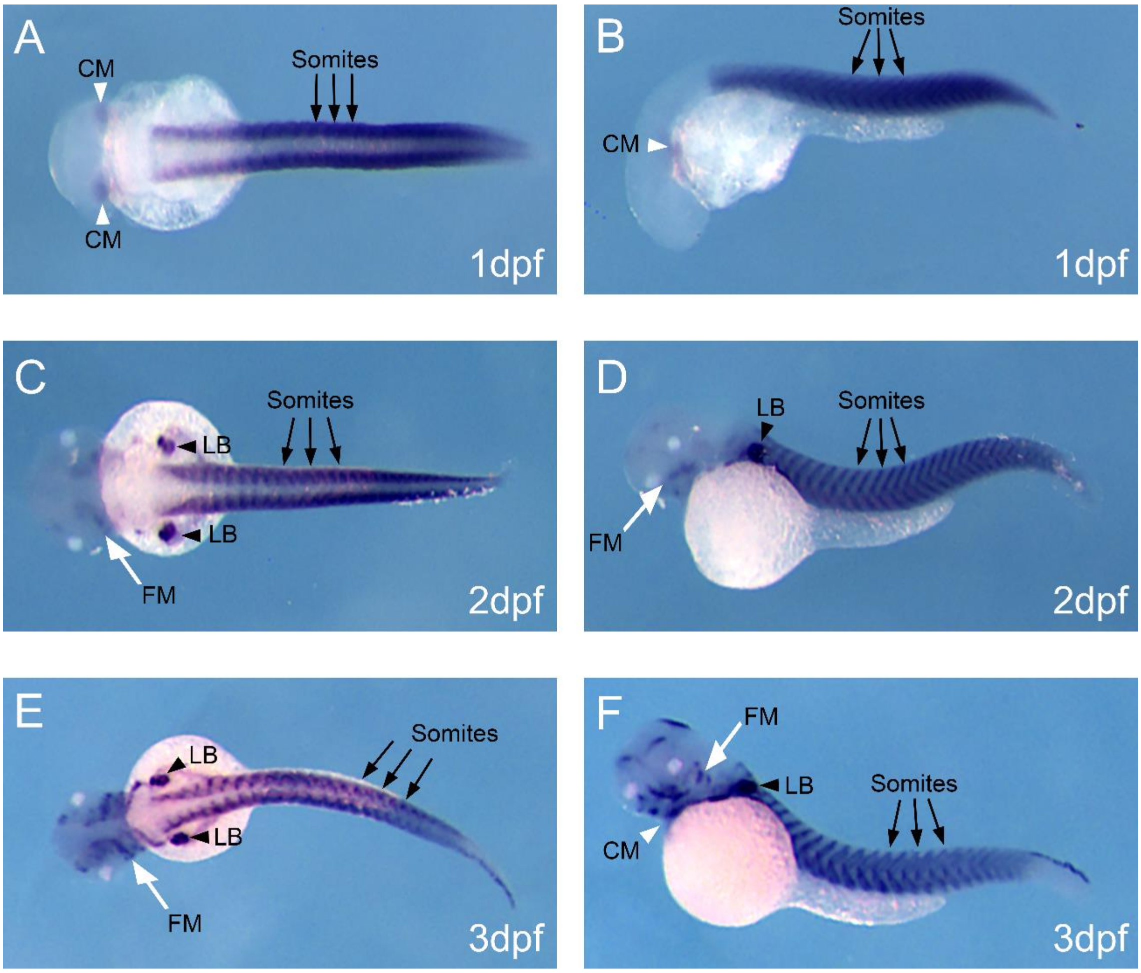

Supplementary Figure 4. Expression of cardiac regulator *mef2ca* in zebrafish.

A-F *in situ* hybridisation of *mef2ca* in zebrafish from 1-3dpf. At 1dpf *mef2ca* is expressed in cardiomyocytes located in bilateral fields of the anterior lateral plate mesoderm (**A, B**). From 1-3dpf, *mef2ca* is expressed in the developing muscle of the somites (**A-F**). At 2dpf and 3dpf *mef2ca* is expressed in the limb bud (**C-F**). At 3dpf *mef2ca* is expressed in the facial muscle (**E, F**). White arrowheads denote cardiomyocytes. Black arrowheads mark limb buds. Black arrows denote somites. White arrows mark and facial muscle. Dorsal views; anterior to the left (**A, C, E**). Lateral views; anterior to the left (**B, D, F**). Abbreviations: CM, cardiomyocytes; LB, limb bud; FM, facial muscle.

Supplementary methods: RNA was extracted from zebrafish embryos at 1-day post fertilisation (dpf) using TRI reagent and used to generate cDNA by reverse transcription. (RT)-PCR was used to amplify *gata4*, *tbx5b* and *mef2ca*. PCR products were ligated into pGEM and used to generate antisense probes for *in situ* hybridisation on zebrafish for comparison with our *O. alcalica* results.

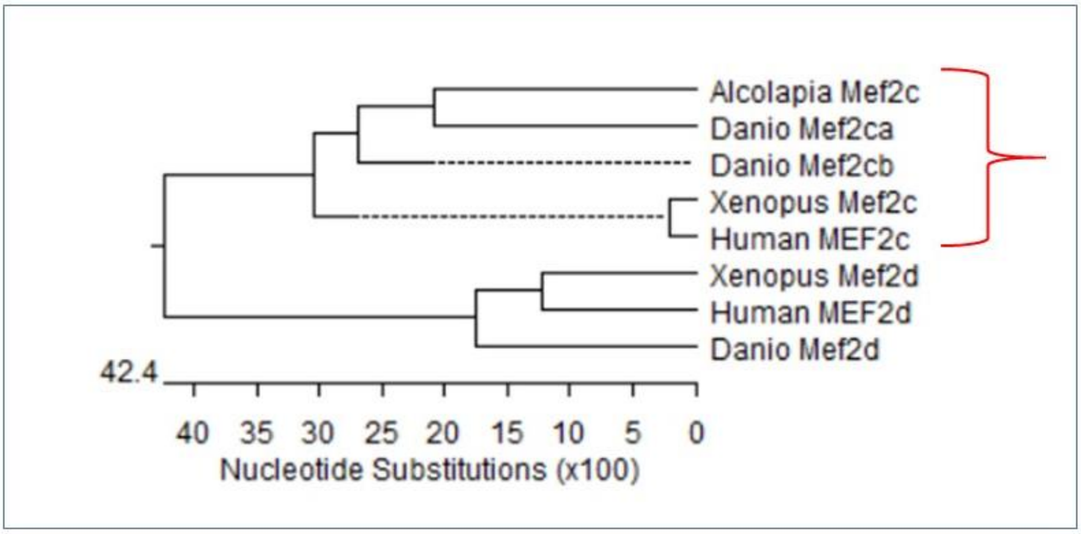

Supplementary Figure 5. Phylogeny of MEFC and MEFD.

Full length protein sequence for *Oreochromis Alcolapia alcalica* Mef2c was aligned to full length protein sequences (obtained from NCBI) of human, Xenopus tropicalis, and Danio rerio Mef2C and Mef2D. The phylogeny clearly shows that the *O.alcalica* Mef2c clades with other Mef2c proteins (red bracket) and not the closely related Mef2d.
